# Single-Crystalline Perovskite Nanowire Arrays for Stable X-ray Scintillators with Micrometer Spatial Resolution

**DOI:** 10.1021/acsanm.1c03575

**Published:** 2021-12-18

**Authors:** Zhaojun Zhang, Hanna Dierks, Nils Lamers, Chen Sun, Klára Nováková, Crispin Hetherington, Ivan G. Scheblykin, Jesper Wallentin

**Affiliations:** † Synchrotron Radiation Research and NanoLund, Department of Physics, Lund University, Box 124, Lund 22100, Sweden; ‡ Chemical Physics and NanoLund, Department of Chemistry, Lund University, Box 124, Lund 22100, Sweden; § Centre for Analysis and Synthesis and NanoLund, Department of Chemistry, Lund University, Box 124, Lund 22100, Sweden

**Keywords:** perovskite, nanowire array, X-ray imaging, micrometer spatial resolution

## Abstract

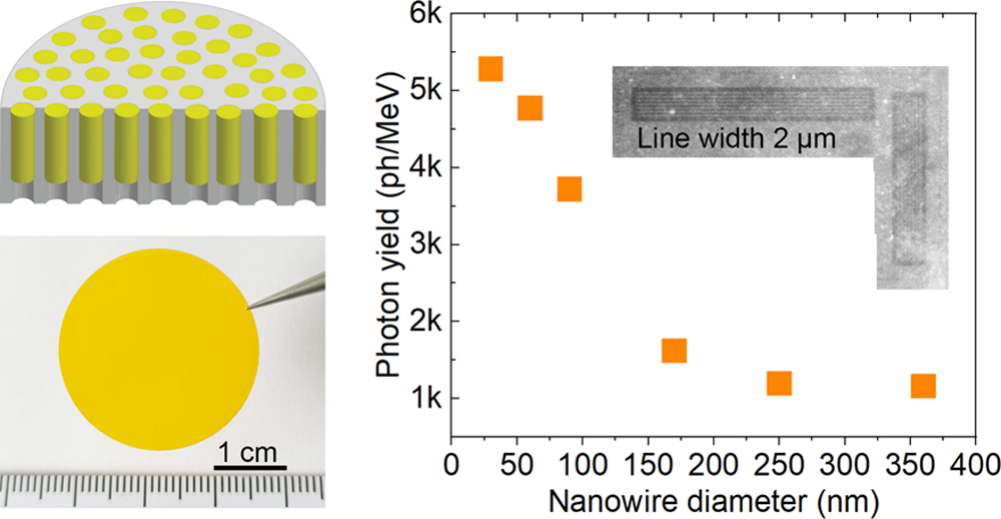

X-ray scintillation detectors based on metal halide perovskites have shown excellent light yield, but they mostly target applications with spatial resolution at the tens of micrometers level. Here, we use a one-step solution method to grow arrays of 15-μm-long single-crystalline CsPbBr_3_ nanowires (NWs) in an AAO (anodized aluminum oxide) membrane template, with nanowire diameters ranging from 30 to 360 nm. The CsPbBr_3_ nanowires in AAO (CsPbBr_3_ NW/AAO) show increasing X-ray scintillation efficiency with decreasing nanowire diameter, with a maximum photon yield of ∼5 300 ph/MeV at 30 nm diameter. The CsPbBr_3_ NW/AAO composites also display high radiation resistance, with a scintillation-intensity decrease of only ∼20–30% after 24 h of X-ray exposure (integrated dose 162 Gy_air_) and almost no change after ambient storage for 2 months. X-ray images can distinguish line pairs with a spacing of 2 μm for all nanowire diameters, while slanted edge measurements show a spatial resolution of ∼160 lp/mm at modulation transfer function (MTF) = 0.1. The combination of high spatial resolution, radiation stability, and easy fabrication makes these CsPbBr_3_ NW/AAO scintillators a promising candidate for high-resolution X-ray imaging applications.

## Introduction

X-ray imaging with micrometer spatial resolution is desirable for the development of applications in physical, materials, and life sciences.^1−3^ High spatial resolution imaging systems for absorption contrast or phase contrast commonly employ indirect detectors that use a scintillator coupled to a CCD or CMOS camera.^4−7^ The scintillator absorbs the X-ray photons and converts them into visible light, which is then focused onto a sensor using a high-resolution objective lens.^7,8^ The key advantage of X-rays is the long penetration length, which allows nondestructive analysis for medical imaging, industrial inspection, etc. However, the long penetration length also makes it challenging to fabricate sensitive X-ray detectors. When the scintillator thickness is increased, the light can spread laterally and reduce the spatial resolution. For an optical detection system with high numerical aperture, the resolution will be limited by the scintillator thickness.^3,7,9,10^ Therefore, thin scintillators are generally used to achieve high spatial resolution, but this limits the absorption and the detection efficiency. The trade-off between sensitivity and resolution makes the fabrication of efficient scintillators for X-ray imaging with micrometer spatial resolution a challenge.

In general, high-performance scintillators need to fulfill several requirements: (1) a composition with heavy elements for strong X-ray absorption, (2) a high scintillation photon yield, (3) an emission spectrum matching the photoresponse of the optical detector, (4) easy and low-cost production, and (5) high stability during storage in ambient conditions and under long-term radiation exposure. Metal halide perovskite (MHP) nanocrystals, such as quantum dots (QDs) and nanosheets, have achieved excellent performance as X-ray scintillators,^11−21^ as they meet the first four earlier-mentioned requirements. However, MHP nanocrystals show a gradual degradation of luminescence in an ambient environment due to reaction with oxygen and water in the air, which is exacerbated by irradiation by UV light or X-rays.^22^ Therefore, current MHP nanocrystal-based scintillators generally use extra protection layers to decrease the degradation.^11,12^ Additionally, the usual thin-film morphology, formed using spin/drop-casting of colloidal nanocrystals, is not beneficial for high spatial resolution imaging due to the lateral scattering of scintillation light. Thus, there is still a lot of room for improvement in both the spatial resolution and the stability of MHP nanocrystal-based X-ray scintillators.

Here, we demonstrate X-ray scintillators based on single-crystalline CsPbBr_3_ nanowire arrays, which address both of these challenges. The nanowires are created by a one-step, low-temperature solution-growth method in commercial anodized aluminum oxide (AAO) membranes. The vertically aligned nanowires reduce the lateral scattering of the scintillation light. Using the CsPbBr_3_ NW/AAO scintillator, X-ray images are able to distinguish line pairs with a spacing of 2 μm, which is significantly better than the tens of micrometers reported previously for MHP nanocrystal film scintillator screens.^12,13,19,21^ The scintillators show an increasing photon yield for decreasing nanowire diameters, with ∼5300 ph/MeV for the smallest 30 nm diameter. Due to the physical confinement of the AAO membrane, our scintillators exhibit significantly improved radiation resistance and air stability. These results, paired with the easy fabrication, make the CsPbBr_3_ NW/AAO scintillators promising for improved X-ray microscopy imaging.

## Results and Discussion

For efficient X-ray detection, the nanowire length should be comparable to the X-ray absorption length in CsPbBr_3_, which is 12 μm for the Cu Kα X-rays (8 keV) used in our experiments. However, growing such long (>10 μm) CsPbBr_3_ nanowires in AAO is challenging. Several studies have demonstrated MHP nanowires in AAO,^23−26^ but the reported nanowire lengths range from hundreds of nanometers to a few micrometers, which is not sufficient for efficient X-ray detection. In our previous study, we showed that the nanowire length can be adjusted by changing the precursor amount or concentration.^26^ The maximum precursor concentration limit is ∼0.45 M in dimethyl sulfoxide (DMSO) for pure phase CsPbBr_3_,^26^ and a continuous supply of precursor is the key challenge for growing longer nanowires. We used 5-μm-thick AAO films on Al substrates, where the pores have only one open end, meaning that the supply of precursor and the evaporation both proceed from the top side of the template. This caused uneven growth of surface solids for nanowire lengths beyond 1–2 μm.

**Figure 1:**
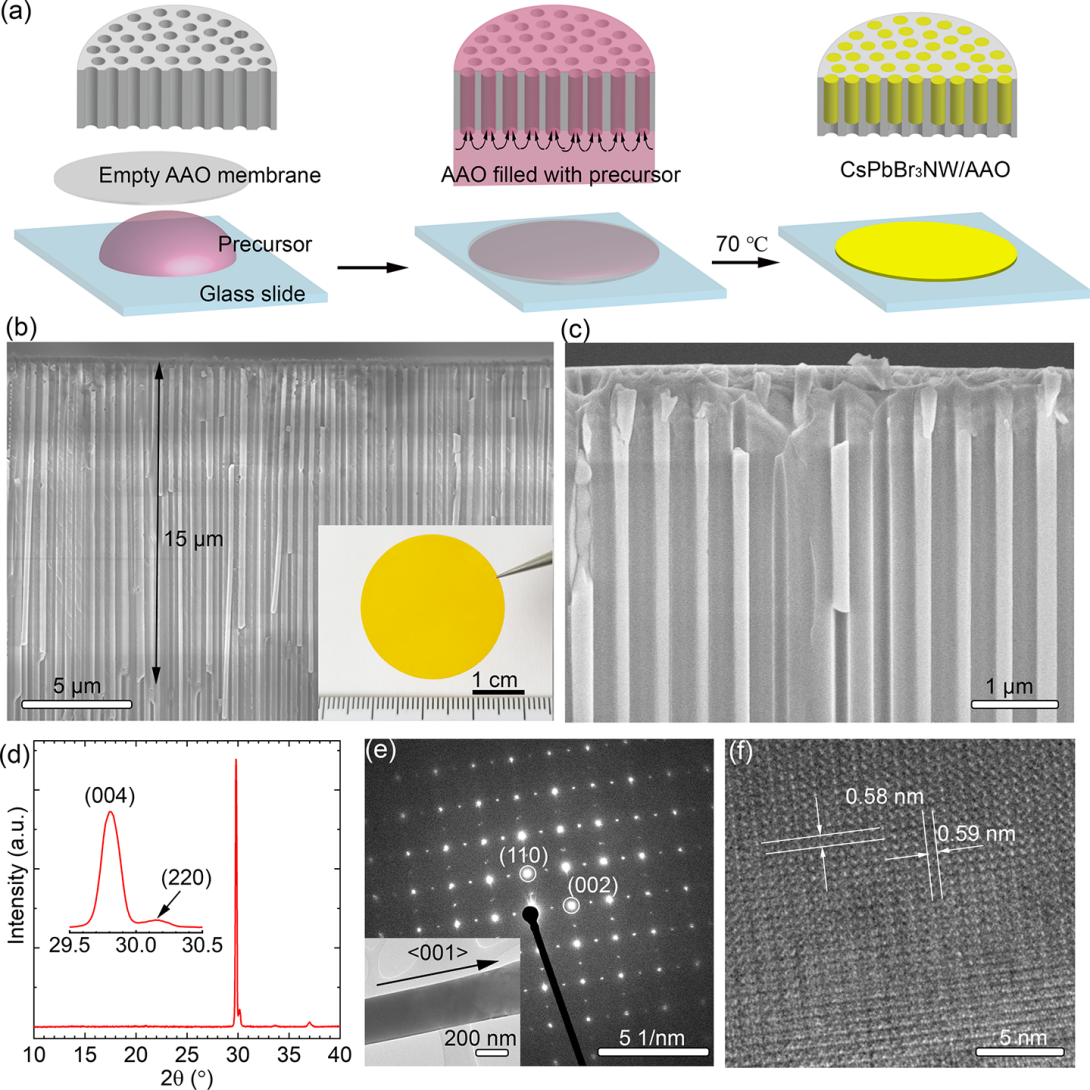
(a) Schematic diagram of the growth process of CsPbBr_3_ NWs in AAO membranes. (b) Cross-sectional scanning electron microscopy (SEM) image showing a uniform nanowire length of ∼15 μm. The inset shows a photo of the as-grown scintillator sample. (c) Higher-magnification SEM showing the nanowires inside the AAO. (d) X-ray diffraction (XRD) pattern of the as-grown sample. The inset shows the 2θ range from 29.5° to 30.5°. (e, f) Transmission electron microscopy (TEM) of a single CsPbBr_3_ nanowire extracted from the cross section of the sample. (e) Selected area electron diffraction (SAED) pattern. The inset shows low-magnification TEM. (f) High-resolution TEM.

In this study, we instead used free-standing AAO membranes, where the nanopores were open at both ends. The growth process of the scintillator is shown in Figure 1a. Briefly, a drop of precursor solution (0.4 M CsPbBr_3_ in DMSO) was put on a glass slide. Then a 50-μm-thick AAO membrane was put on top of the precursor droplet. After waiting for 1 min to make sure the AAO pores were filled with precursor by capillary forces, the sample was transferred to a hot plate and held at 70 °C for 30 min until all of the solvent had evaporated. As seen in Figure 1a, the evaporation proceeded from the top side. During the evaporation process, as the precursor inside the pores was consumed, the liquid precursor on the bottom side continuously entered the pores by capillary forces. Because most of the precursor was kept under the bottom of the AAO, the formation of surface solids was significantly decreased compared with our previous report.^26^ Using this method, as seen in the cross-sectional SEM image in Figure 1b, we successfully achieved a nanowire length of 15 μm inside the 50-μm-thick AAO membrane. The samples had a uniform color, as shown in the inset photograph, which indicated homogeneous growth and a clean surface. We systematically studied the influence of the nanowire diameter for the scintillator properties by using AAO templates with different pore diameters. Here, we will first show in-depth characterization of the nominal 170-nm-diameter samples, while the diameter dependence of the nanowires will be discussed in the following part. At the end, the X-ray imaging characterization is displayed and analyzed.

The high-magnification cross-sectional SEM image in Figure 1c shows the vertical alignment of the nanowires inside the pores. As seen in Figure S1 in the Supporting Information, energy-dispersive X-ray spectroscopy (EDS) showed a uniform distribution of Cs, Pb, and Br elements with an element ratio of ∼1:1:3.4 (Cs/Pb/Br), which agreed closely with the stoichiometric ratio of CsPbBr_3_. X-ray diffraction (XRD) patterns of the as-grown membrane are shown in Figure 1d. The splitting of the (004) and (220) peaks fit the expected orthorhombic phase CsPbBr_3_ (*pnma*, *a* = 8.207 Å, *b* = 8.255 Å, and *c* = 11.759 Å).^27,28^ However, while a random powder had a relative peak intensity (004)/(220) of ∼0.6:1, we found a ratio of 12:1 due to nanowire growth along the <001> direction and the excellent vertical alignment of the nanowires.

To examine the crystalline quality and growth direction of the nanowires, we used a focused ion beam (FIB) probe to extract a single nanowire (Figure 1e) from the AAO pores for transmission electron microscopy (TEM) measurements. The selected area electron diffraction (SAED) pattern in Figure 1e confirmed that the nanowire was single crystalline and it had grown along the <001> direction. High-resolution TEM (HRTEM), as shown in Figure 1f, gave a spacing of 0.59 nm for the (002) planes and 0.58 nm for the (110) planes, which was in agreement with the XRD measurement. Thus, the structural analysis demonstrated the vertical alignment and single-crystal nature of the CsPbBr_3_ nanowires. The whole sample was an array of single-crystalline CsPbBr_3_ nanowires formed inside an AAO membrane, and it is referred to as CsPbBr_3_ NW/AAO in the following text.

**Figure 2:**
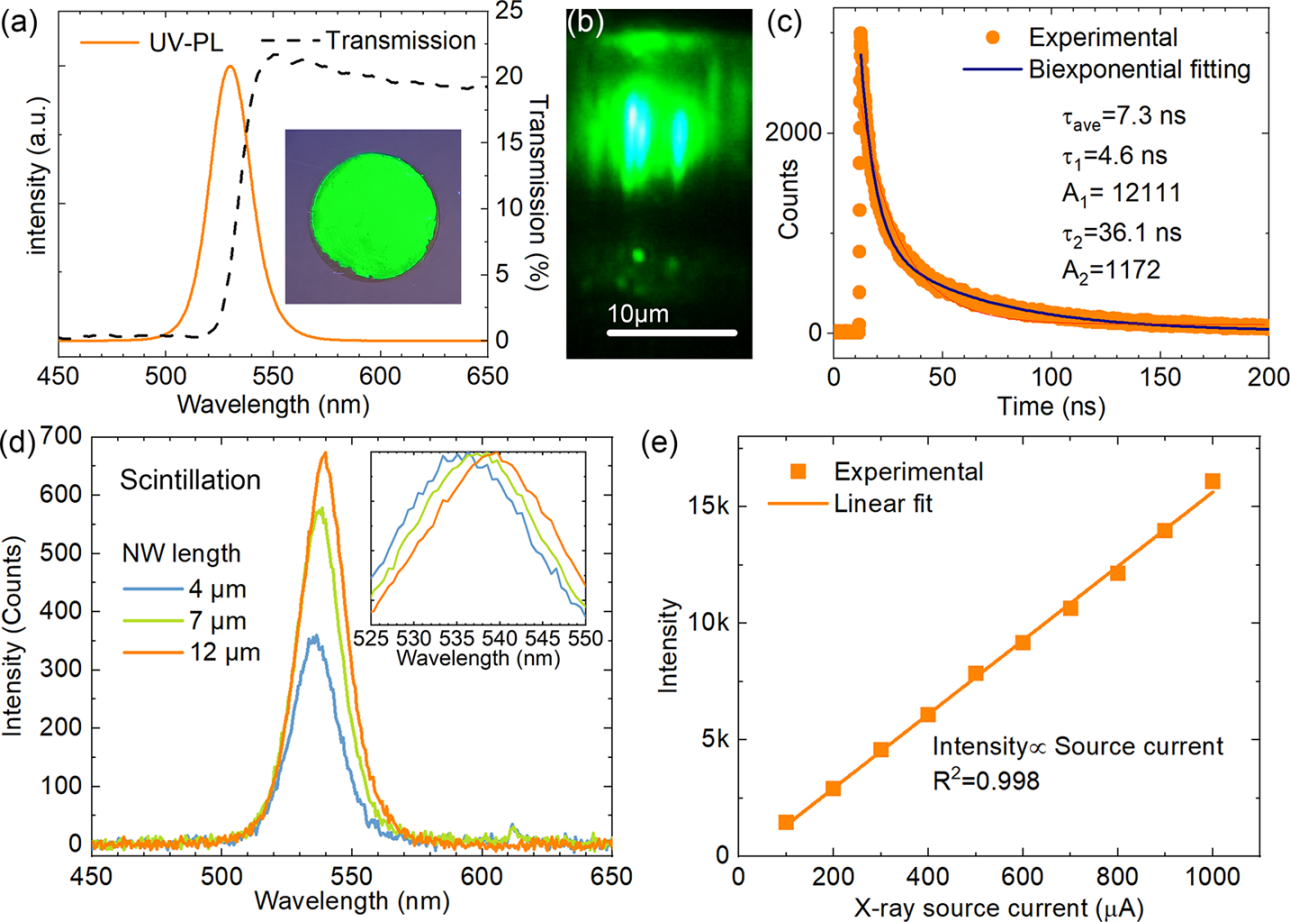
Optical properties of the CsPbBr_3_ NW/AAO samples with a nominal diameter of 170 nm. (a) Transmission spectrum (dashed line) and photoluminescence (PL) spectrum (solid line) when excited by a 378 nm UV laser (continuous-wave mode). The inset shows the entire sample illuminated by a 365-nm UV flashlight. (b) PL microscopy image of a cross-section, showing light guiding from the central excitation spot to the nanowire tips (excitation: 378 nm laser). (c) Time-resolved PL decay profile (excitation: 485 nm pulsed laser with excitation power density of 110 mW/cm^2^ and repetition rate 350 kHz). (d) Scintillation spectra (X-ray source 40 kV, 1 mA, 1.88 mGy_air_/s) for different lengths of nanowires. The inset shows the normalized spectra in a smaller range, from 525 to 550 nm. (e) Integrated scintillation intensity versus the X-ray source current (40 kV, 100 μA–1 mA).

The CsPbBr_3_ NW/AAO structures are interesting for a wide range of applications, and in most cases, such as X-ray scintillators, the optical properties are essential.^26^Figure 2a displays the transmission spectrum and ultraviolet (UV) laser-excited photoluminescence (PL) spectrum of the CsPbBr_3_ NW/AAO membrane. The inset photo shows the strong and uniform green luminescence of the sample under irradiation with a 365 nm UV flashlight. The transmission spectrum had an absorption edge at ∼540 nm, and the UV-PL emission peak was positioned at 530 nm. These values were consistent with the values in previous studies of CsPbBr_3_ nanowires.^28−30^

The nanowires inside the AAO pores displayed clear light guiding, as observed in Figure 2b, which was in line with previous reports of light guiding behavior of single CsPbBr_3_ nanowires or microwires.^31−35^ Note that the light detection in this geometry, orthogonal to the nanowire axis, was perpendicular to how the light was detected in the actual scintillator. Therefore, it was reasonable to assume that the guided light was preferentially emitted along the nanowire axis. The light guiding could help reduce the lateral spread of the scintillation light, which would be useful for achieving high spatial resolution X-ray imaging without sacrificing the thickness of the scintillator.^9,36−38^ Additionally, this kind of vertical nanowire array may also have applications in other optoelectronic fields such as photovoltaics, due to their potential efficient light management (enhanced optical absorption, light guiding, etc.).^39^

We investigated the UV laser-excited time-resolved PL decay profile, as shown in Figure 2c, and fitted the decays with a biexponential function . The fitting indicated that there were two decay channels with lifetimes of τ_1_ = 4.6 ns and τ_2_ = 36 ns, respectively,^40,41^ similar to previously reported decay lifetimes in CsPbBr_3_ nanowires.^33^ The calculated amplitude-averaged lifetime, τ_ave_ = , was ∼7.3 ns.

Figure 2d presents the scintillation (X-ray-excited luminescence) spectra for different lengths of CsPbBr_3_ nanowires in AAO. The length of the nanowires was adjusted by changing the precursor amounts (details are in the Experimental Section in the Supporting Information). The luminescence intensity increased with the length, as expected, due to the increased X-ray absorption. The peak position showed a small but clear red-shift with increasing nanowire length, which could be attributed to reabsorption of the short-wavelength part of the spectrum.^34,39^ The reabsorption of the shorter-wavelength emission was more efficient. Additionally, compared to Figure 2a, the X-ray luminescence peak position displayed a red-shift compared to the UV-PL, which could also be due to the reabsorption effect because the penetration of the X-rays (12 μm for the Cu Kα) was much larger than that of the UV laser. The different excitation conditions could also contribute to the peak position shift between UV- and X-ray-excited luminescence. Figure 2e displays how the scintillation intensity scaled with the X-ray source current, demonstrating a linear relationship. Note that the X-ray photon flux also increased linearly with the source current, as shown in Figure S2. This linearity is an important property for the practical applications for X-ray imaging.

The nanowire diameter was previously shown to affect optical properties such as PL intensity and peak position.^26^ We made CsPbBr_3_ NWs in AAO membranes having similar lengths but with pore diameters of 30 (±5), 60 (±10), 90 (±10), 170 (±30), 250 (±30), and 360 (±40) nm, using the same precursor concentration and amount. These samples are labeled D30, D60, etc. in the following text. Photos and SEM images of all of the samples are shown in Figure S3, cross-sectional SEM images are shown in Figure S4, and XRD measurements of all of the samples are shown in Figure S5.

**Figure 3:**
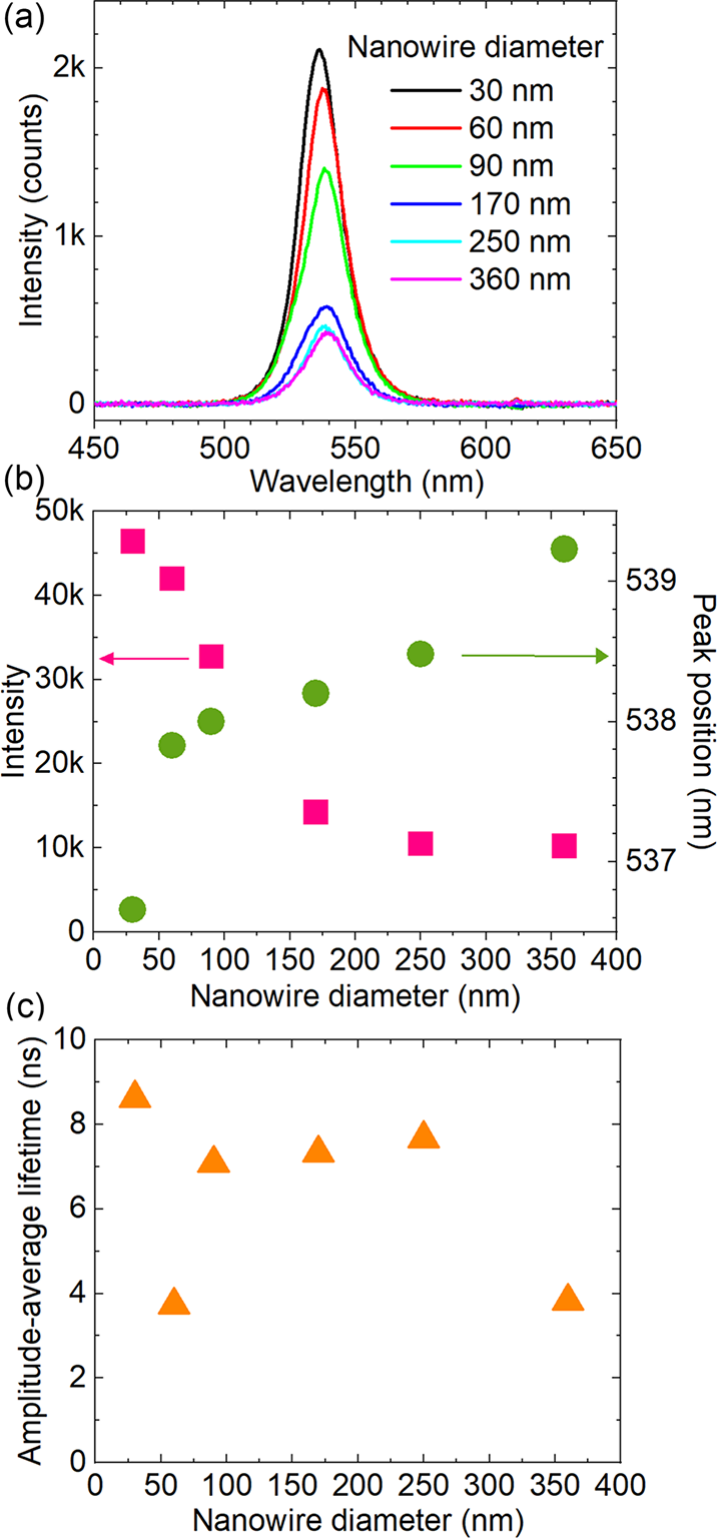
(a) Scintillation spectra for different nanowire diameters. The nanowire length is ∼7 μm. X-ray source = 45 kV, 1 mA, 1.88 mGy_air_/s. (b) Diameter dependence of the peak position (green circles, right) and integrated luminescence intensity (pink squares, left) extracted from (a). (c) Average time-dependent PL decay lifetimes vs nanowire diameter.

The scintillation spectra for CsPbBr_3_ nanowires with different diameters are displayed in Figure 3a, while the luminescence intensity and the peak position from peak fitting are shown in Figure 3b. Surprisingly, the X-ray luminescence spectra showed an increasing luminescence intensity for smaller diameters, where the D30 sample had 5 times stronger scintillation intensity than the D360 sample. As a further comparison, we also grew a CsPbBr_3_ thin film with a thickness of ∼40 μm (Figure S6). All of the CsPbBr_3_ NW/AAO samples exhibited a much higher X-ray luminescence intensity than the significantly thicker CsPbBr_3_ film. We also observed a slight blue-shift of the emission wavelength with decreasing diameter. The trends were consistent with the UV-excited PL spectra, as shown in Figure S7. The slight blue-shift of the X-ray and UV laser-excited emission for the thinner nanowires could be due to several reasons, as discussed in our previous report.^26^ The smaller diameter nanowires had a larger distortion of the lattice, which may affect the band structure,^42^ and the strain in the nanowire that resulted from the confinement inside the AAO also affected the emission wavelength.^43^ Additionally, the Stokes shift and self-absorption effects in thicker nanowires may have contributed.^30,44^

To measure the photon yield of different diameter CsPbBr_3_ NW/AAO samples, we used a commercial YAG:Ce scintillator with 500-μm thickness as a reference (X-ray luminescence spectrum in Figure S8). In line with the earlier results, the smallest-diameter (D30) CsPbBr_3_ NW/AAO sample had the highest photon yield of ∼5 300 ph/MeV. This value was comparable to commercial YAP:Pr (6 000 ph/MeV) and BGO (Bi_4_Ge_3_O_12_, 8 000 ph/MeV) scintillators but lower than commercial YAG:Ce (30 000 ph/MeV), CsI(Tl) (54 000 ph/MeV), and Gadox (Gd_2_O_2_S:Tb, 65 000 ph/MeV).^45,46^ The X-ray absorption in the D30 CsPbBr_3_ NW/AAO scintillator, as measured with a calibrated diode, was 31%. It should be noted that the CsPbBr_3_ nanowires were grown inside the AAO, where the aluminum oxide itself absorbed a significant share of the X-rays without any scintillation output. If we compensated for the X-ray photons absorbed by AAO, as explained in the Supporting Information, the estimated net photon yield of the 30 nm-diameter CsPbBr_3_ nanowires was ∼19 000 ph/MeV, which was comparable to the previously reported photon yield of a CsPbBr_3_ QD film (21 000 ph/MeV)^47^ and ∼2 orders of magnitude more than our CsPbBr_3_ microcrystal film (∼200 ph/MeV).

Additionally, we measured the UV laser-excited time-resolved PL decay profiles of all of the different diameter CsPbBr_3_ NW/AAO samples in Figure S9 (excitation: 485 nm pulsed laser). All of the decay profiles were fitted with a biexponential function, as discussed earlier, and the calculated amplitude-average lifetimes for different diameters CsPbBr_3_ NW/AAO are shown in Figure 3c. The decays were significantly faster than those for commercial scintillators such as YAG:Ce (90–100 ns), NaI:Tl (230 ns), etc.,^15,45^ which could make our CsPbBr_3_ NW/AAO scintillators promising for ultrafast X-ray detection.

The observed photon yield of CsPbBr_3_ NW/AAO, from both UV and X-ray excitation, increased with decreasing nanowire diameter. This was unexpected because the thinner nanowires had a much higher surface-to-volume ratio in comparison with the thicker ones, which, in principle, should have increased surface recombination. There are several possible explanations for the increased photon yield: (1) The AAO could passivate the nanowires, instead of causing defects for nonradiative surface recombination.^48^ (2) The interface could have states that are favorable, not detrimental, for the luminescence efficiency.^11^ (3) The decrease of the nanowire diameter increases the exciton binding energy, which is favorable for reaching higher luminescence efficiency. Gao et al. reported that 15-nm-thick CsPbBr_3_ nanowires had an exciton binding energy of 93 meV, compared with 65 meV for the 250-nm-diameter ones.^30^ (4) The dielectric confinement causes an increased luminescence efficiency. Lin et al. reported that the dielectric confinement induced a high emission quantum yield of tin perovskites.^49^ The decrease of the diameter possibly leads to an increase of the dielectric constant of the CsPbBr_3_ nanowires.^35^ (5) There could be reduced light reabsorption. The smaller-diameter AAO membranes had higher transmittance for the 520–540 nm light (see transmission spectra of different pore diameter empty AAO membranes—i.e., without nanowires—in Figure S10). In addition, when the diameter of the nanowires became much smaller than the wavelength, waveguiding along the wires was reduced, decreasing the interaction length of the luminescence light with the perovskite material and improving the light outcoupling.

**Figure 4:**
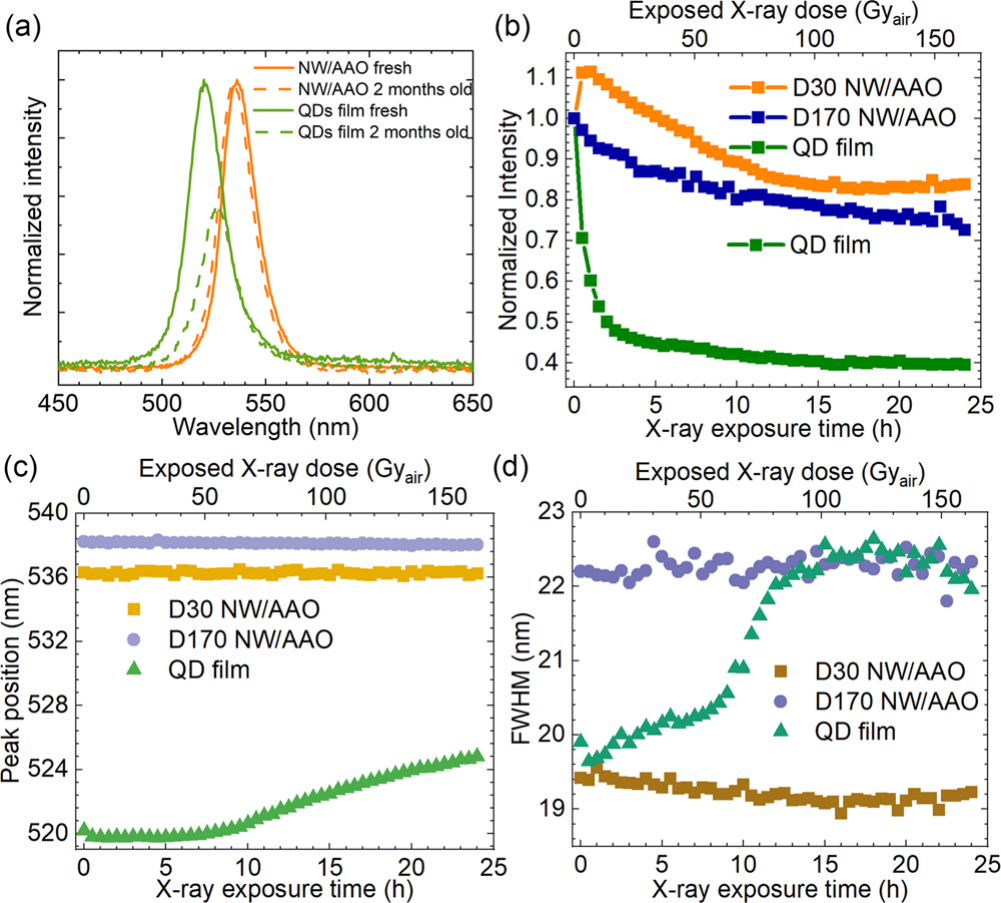
(a) Scintillation spectra of fresh and 2-month-old CsPbBr_3_ NW/AAO and CsPbBr_3_ QD films. (b) Relative intensity, (c) peak position, and (d) full width at half-maximum (fwhm) evolutions for CsPbBr_3_ NW/AAO samples and the CsPbBr_3_ QD film during a 24-h X-ray exposure (45 kV, 1 mA, 6.77 Gy_air_/h).

One of the main challenges with the use of MHP in applications is their degradation with exposure to moisture, oxygen, light, and X-rays.^50^ Generally, CsPbBr_3_ QD films need to be protected to decrease their degradation under ambient conditions or light irradiation. For our CsPbBr_3_ NW/AAO membrane scintillators, the CsPbBr_3_ nanowires are grown inside the AAO, which can act as a shield to protect them from degradation.^26^Figure 4a displays the scintillation spectra of a freshly grown and a two month old CsPbBr_3_ NW/AAO membrane scintillator. The spectra are almost identical, which demonstrates a high stability of CsPbBr_3_ NW/AAO under ambient storage conditions. In comparison, the CsPbBr_3_ QD film shows an almost 50% decrease in the intensity after two months under the same storage conditions. Therefore, it is concluded that the physical confinement provided by the AAO improved the stability of CsPbBr_3_ nanowires, which can be attributed to the effective prevention of water and oxygen to diffuse into the sidewalls of the nanowires.^48^

Radiation resistance is obviously a crucial feature for a scintillator. Therefore, we measured the scintillation spectra under continuous 24 h X-ray exposure, using a CsPbBr_3_ QD film sample as comparison. The measurements were done in air. As shown in Figure 4b, the signal from the QD film rapidly decreased by 30% after 0.5 h, 50% after 2 h, and 60% after 24 h. In comparison, the D30 and D170 CsPbBr_3_ NW/AAO samples displayed 20% and 30% decreases after 24 h exposure, respectively. Additionally, the D30 CsPbBr_3_ NW/AAO showed an initial 10% increase of the intensity after 0.5–1 h X-ray exposure, followed by a gradual decrease for 15 h and finally a plateau. The scintillation intensity increase could possibly be due to defect curing by oxygen.^51,52^ We observed a similar phenomenon in our previous study of D20 CsPbBr_3_ NW/AAO on Al substrates.^26^ This phenomenon was not observed in the D170 CsPbBr_3_ NW/AAO. A possible explanation is that the surface defect states that can be cured are more significant for the thinner nanowires. The peak position and width of the CsPbBr_3_ NW/AAO sample were quite stable even after 24 h exposure, as shown in Figure 4c, while the QD film had an evident red-shift of peak position and peak broadening. In CsPbBr_3_ QDs, the emission wavelength showed a blue-shift compared with the NWs due to quantum confinement. We speculate that long-term X-ray exposure caused aggregation of the QDs in the film, which led to a red-shift and peak broadening of the emission peak position. This kind of aggregation was obviously impossible for the CsPbBr_3_ nanowires inside the AAO nanopores.

**Figure 5:**
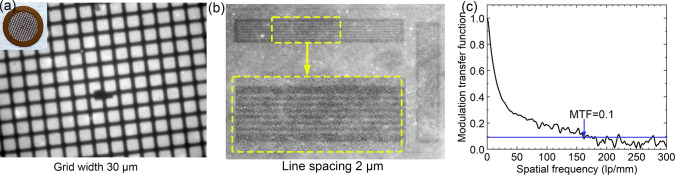
(a) X-ray image of a TEM grid using the CsPbBr_3_ NW/AAO scintillator. The width of the bars is 30 μm (X-ray: 45 kV, 1 mA, 0.7 Gy_air_/h). (b) X-ray image of a JIMA test pattern with a line spacing of 2 μm (X-ray: 45 kV, 1 mA, 0.7 Gy_air_/h). (c) Modulation transfer function of the imaging by slant-edge method (X-ray: 45 kV, 1 mA, 0.7 Gy_air_/h).

To test the X-ray imaging performance of our scintillator, we used a TEM stainless steel grid with a thickness of 12 μm and a bar width of 30 μm as a test sample. As displayed in Figure 5a, the final image had good contrast. To examine the spatial resolution that our CsPbBr_3_ NW/AAO scintillators can reach, we imaged a JIMA resolution test pattern. Because our X-ray source was a Cu target and the absorption length of Cu Kα in CsPbBr_3_ was ∼12 μm, we used samples with a nanowire length of ∼12–13 μm. All of the different diameter samples had a similar length, as the cross-sectional SEM images show in Figure S11. The smallest line space that could be distinguished was 2 μm, corresponding to 250 lp/mm, as shown in Figure 5b. All of the different diameter samples had the same spatial resolution (Figure S12) but different brightnesses due to their different photon yields, as discussed earlier. Additionally, we measured the MTF using a slanted-edge method (Figure 5c), which showed a spatial resolution of ∼160 lp/mm at a contrast of 0.1, which corresponded to a line spacing of ∼3.1 μm. This was close to the result obtained from the JIMA pattern measurement. The slight difference could be from the different evaluation mechanisms of these two methods. We were not able to measure the resolution of the thin-film sample, due to the weak signal, or of the QD sample, due to an uneven deposition thickness.

As compared in Table 1, the spatial resolution of our CsPbBr_3_ NW/AAO scintillator was slightly better than those for previously reported non-MHP materials with vertically aligned columnar structures such as ZnO nanowires (3 μm)^5^ and a CsI (Tl) needle/Si array (5 μm).^37^ Compared to previously reported CsPbBr_3_ or MAPbBr_3_ nanocrystal film-based scintillators,^12,13,16,19,21^ our scintillator was significantly better. Very recently, Li et al. reported scintillators with a columnar structure, using AAO templates filled with CsPbBr_3_ QDs, and observed a line spacing of 2 μm for JIMA patterns with a synchrotron X-ray source.^18^ Note that many previous reports used higher X-ray energy and thicker scintillators, which was detrimental to the spatial resolution. We believe that one important reason for the high spatial resolution was that the CsPbBr_3_ NW/array structure decreased the lateral spread of the scintillation light.^10^ Wang et al. used a CsPbBr_3_ nanosheet film with a thickness of 15 μm as the X-ray scintillator and achieved 26-μm resolution.^19^ Because this film thickness was almost the same as our nanowire length, the higher spatial resolution in our results indicated that the columnar nanowire structure was indeed beneficial for the spatial resolution.

**Table 1:** Comparison of X-ray Imaging Spatial Resolution of Our Results with Previously Reported MHP Scintillators and Other Nanowire-Based Scintillators from Other Materials^a^

materials	spatial resolution, MTF (lp/mm)	spatial resolution, line spacing (μm)	thickness	methods	ref
ZnO nanowires		3	200 nm	electrodeposition	(5)
CsPbBr_3_ nanocrystal	12.5		1.62 mm	spin-coating	(12)
CsPbBr_3_ nanosheet	2.4		25 μm	drop-casting	(13)
CsPbBr_3_ nanocrystal	16.8		0.8 mm	melting quenching	(16)
CsPbBr_3_ QDs/AAO	211	2	20 μm	pressure filling	(18)
CsPbBr_3_ nanosheet		26	15 μm	self-assembly	(19)
MAPbBr_3_ QD film	5.3		50 μm	spin-coating	(21)
CsI (Tl) needle/Si	100	5	40 μm	melt-filling	(37)
CsPbBr_3_ NW/AAO	160	2	12–13 μm	solution	this work

a The spatial resolution is divided into two parts based on whether the results shown in the literature were acquired by the MTF function or line spacing.

As shown in Figure 2b, we observed light-guiding in the D170 CsPbBr_3_ NW/AAO, and we also observed this in the D250 and D360 CsPbBr_3_ NW/AAO. However, when the nanowire diameter (D30, D60, and D90) was much smaller than the scintillation light wavelength (536–540 nm in our results), the light-guiding was not clearly observed. The reason could be a reduced confinement in the thinner nanowires, but it is also because it was experimentally more difficult to observe this effect for such thin and dense nanowires. Therefore, for the smaller-diameter nanowires, there might be a different cause of the excellent spatial resolution compared with the larger-diameter samples. Generally, scintillators had increased spatial resolution with decreasing film thickness due to the reduced transversal light scattering.^7,10^ For higher-energy X-rays used in clinical applications,^53,54^ nanowires with lengths of up to a few hundred micrometers are needed, and we believe that the light-guiding advantages of the CsPbBr_3_ NW/AAO scintillators will be comparatively more useful in this case.

## Conclusion

In this work, we designed a straightforward one-step solution method to grow single-crystal CsPbBr_3_ nanowires vertically aligned inside AAO membranes. Low-temperature solution growth offers lower cost and higher scalability than more complex synthesis methods, and it could be extended to other MHPs. The CsPbBr_3_ NW/AAO composites showed increased X-ray luminescence photon yield with decreasing diameter, where the 30-nm-diameter NW/AAO had a photon yield of 5 300 ph/MeV. Benefiting from the physical confinement of AAO, the scintillators showed high resistance to continuous X-ray radiation and ambient condition storage. Using the CsPbBr_3_ NW/AAO scintillator, X-ray imaging with ∼2 μm spatial resolution was demonstrated. The MHP NW/AAO materials are promising for applications in X-ray imaging with micrometer-level spatial resolution, and the improved stability makes them interesting for other optoelectronic applications.

## Supporting Information Available

Detailed materials growth and characterization, EDS mapping results of nanowires inside AAO, relation of X-ray photon flux to the source current, photos and top-view and cross-sectional view SEM images, XRD patterns, UV-excited luminescence spectra, time-resolved PL decay, X-ray imaging results of different diameter CsPbBr_3_ NW/AAO samples, transmission spectra of different pore diameter empty AAO, and scintillation spectra of YAG:Ce scintillator and CsPbBr_3_ microcrystal film (PDF)

## References

[ref1] Schunck J. O.; Döring F.; Rösner B.; Buck J.; Engel R. Y.; Miedema P. S.; Mahatha S. K.; Hoesch M.; Petraru A.; Kohlstedt H.; Schüssler-Langeheine C.; Rossnagel K.; David C.; Beye M. Soft X-ray imaging spectroscopy with micrometer resolution. Optica 2021, 8 (2), 156–160. 10.1364/OPTICA.405977.

[ref2] Borisova E.; Lovric G.; Miettinen A.; Fardin L.; Bayat S.; Larsson A.; Stampanoni M.; Schittny J. C.; Schlepütz C. M. Micrometer-resolution X-ray tomographic full-volume reconstruction of an intact post-mortem juvenile rat lung. Histochem. Cell Biol. 2021, 155 (2), 215–226. 10.1007/s00418-020-01868-8. 32189111 PMC7910225

[ref3] Martin T.; Koch A. Recent developments in X-ray imaging with micrometer spatial resolution. J. Synchrotron Radiat. 2006, 13 (2), 180–194. 10.1107/S0909049506000550. 16495618

[ref4] Larsson J. C.; Lundström U.; Hertz H. M. Characterization of scintillator-based detectors for few-ten-keV high-spatial-resolution x-ray imaging. Med. Phys. 2016, 43 (6), 2731–2740. 10.1118/1.4948687. 27277020

[ref5] Kobayashi M.; Komori J.; Shimidzu K.; Izaki M.; Uesugi K.; Takeuchi A.; Suzuki Y. Development of vertically aligned ZnO-nanowires scintillators for high spatial resolution x-ray imaging. Appl. Phys. Lett. 2015, 106 (8), 081909 10.1063/1.4913867.

[ref6] Touš J.; Horváth M.; Pína L.; Blažek K.; Sopko B. High-resolution application of YAG:Ce and LuAG:Ce imaging detectors with a CCD X-ray camera. Nucl. Instrum. Methods Phys. Res., Sect. A 2008, 591 (1), 264–267. 10.1016/j.nima.2008.03.070.

[ref7] Rocha J. G.; Lanceros-Mendez S. Review on X-ray detectors based on scintillators and CMOS technology. Recent Pat. Electr. Eng. 2011, 4 (1), 16–41. 10.2174/1874476111104010016.

[ref8] Lin R.; Zheng W.; Chen L.; Zhu Y.; Xu M.; Ouyang X.; Huang F. X-ray radiation excited ultralong (>20,000 seconds) intrinsic phosphorescence in aluminum nitride single-crystal scintillators. Nat. Commun. 2020, 11 (1), 4351 10.1038/s41467-020-18221-1. 32859949 PMC7455697

[ref9] Miller S. R.; Gaysinskiy V.; Shestakova I.; Nagarkar V. V. Recent advances in columnar CsI(Tl) scintillator screens; SPIE: 2005; Vol. 5923.

[ref10] Luo Z.; Moch J. G.; Johnson S. S.; Chen C. C. A Review on X-ray Detection Using Nanomaterials. Curr. Nanosci. 2017, 13 (4), 364–372. 10.2174/1573413713666170329164615.

[ref11] Chen Q.; Wu J.; Ou X.; Huang B.; Almutlaq J.; Zhumekenov A. A.; Guan X.; Han S.; Liang L.; Yi Z.; Li J.; Xie X.; Wang Y.; Li Y.; Fan D.; Teh D. B. L.; All A. H.; Mohammed O. F.; Bakr O. M.; Wu T.; Bettinelli M.; Yang H.; Huang W.; Liu X. All-inorganic perovskite nanocrystal scintillators. Nature 2018, 561 (7721), 88–93. 10.1038/s41586-018-0451-1. 30150772

[ref12] Heo J. H.; Shin D. H.; Park J. K.; Kim D. H.; Lee S. J.; Im S. H. High-Performance Next-Generation Perovskite Nanocrystal Scintillator for Nondestructive X-Ray Imaging. Adv. Mater. 2018, 30 (40), 1801743 10.1002/adma.201801743. 30141200

[ref13] Zhang Y.; Sun R.; Ou X.; Fu K.; Chen Q.; Ding Y.; Xu L.-J.; Liu L.; Han Y.; Malko A. V.; Liu X.; Yang H.; Bakr O. M.; Liu H.; Mohammed O. F. Metal Halide Perovskite Nanosheet for X-ray High-Resolution Scintillation Imaging Screens. ACS Nano 2019, 13 (2), 2520–2525. 10.1021/acsnano.8b09484. 30721023

[ref14] Wang L.; Fu K.; Sun R.; Lian H.; Hu X.; Zhang Y. Ultra-stable CsPbBr_3_ Perovskite Nanosheets for X-Ray Imaging Screen. Nano-Micro Lett. 2019, 11 (1), 52 10.1007/s40820-019-0283-z. PMC7770729 34138025

[ref15] Cao F.; Yu D.; Ma W.; Xu X.; Cai B.; Yang Y. M.; Liu S.; He L.; Ke Y.; Lan S.; Choy K.-L.; Zeng H. Shining Emitter in a Stable Host: Design of Halide Perovskite Scintillators for X-ray Imaging from Commercial Concept. ACS Nano 2020, 14 (5), 5183–5193. 10.1021/acsnano.9b06114. 31774652

[ref16] Zhang H.; Yang Z.; Zhou M.; Zhao L.; Jiang T.; Yang H.; Yu X.; Qiu J.; Yang Y.; Xu X. Reproducible X-ray Imaging with a Perovskite Nanocrystal Scintillator Embedded in a Transparent Amorphous Network Structure. Adv. Mater. 2021, 33 (40), 2102529 10.1002/adma.202102529. 34418177

[ref17] Maddalena F.; Xie A.; Chin X. Y.; Begum R.; Witkowski M. E.; Makowski M.; Mahler B.; Drozdowski W.; Springham S. V.; Rawat R. S.; Mathews N.; Dujardin C.; Birowosuto M. D.; Dang C. Deterministic Light Yield, Fast Scintillation, and Microcolumn Structures in Lead Halide Perovskite Nanocrystals. J. Phys. Chem. C 2021, 125 (25), 14082–14088. 10.1021/acs.jpcc.1c03392.

[ref18] Li H.; Yang H.; Yuan R.; Sun Z.; Yang Y.; Zhao J.; Li Q.; Zhang Z. Ultrahigh Spatial Resolution, Fast Decay, and Stable X-Ray Scintillation Screen through Assembling CsPbBr_3_ Nanocrystals Arrays in Anodized Aluminum Oxide. Adv. Opt. Mater. 2021, 2101297 10.1002/adom.202101297.

[ref19] Wang Z.; Sun R.; Liu N.; Fan H.; Hu X.; Shen D.; Zhang Y.; Liu H. X-Ray imager of 26-μm resolution achieved by perovskite assembly. Nano Res. 2021, 10.1007/s12274-021-3808-y

[ref20] Maddalena F.; Xie A.; Arramel; Witkowski M. E.; Makowski M.; Mahler B.; Drozdowski W.; Mariyappan T.; Springham S. V.; Coquet P.; Dujardin C.; Birowosuto M. D.; Dang C. Effect of commensurate lithium doping on the scintillation of two-dimensional perovskite crystals. J. Mater. Chem. C 2021, 9 (7), 2504–2512. 10.1039/D0TC05647B.

[ref21] Xu Q.; Zhou S.; Huang J.; Ouyang X.; Liu J.; Guo Y.; Wang J.; Nie J.; Zhang X.; Ouyang X.; Jia W. Ultra-flexible and highly sensitive scintillation screen based on perovskite quantum dots for non-flat objects X-ray imaging. Materials Today Physics 2021, 18, 100390 10.1016/j.mtphys.2021.100390.

[ref22] Soosaimanickam A.; Rodríguez-Cantó P. J.; Martínez-Pastor J. P.; Abargues R. Chapter 4: Surface modification of all-inorganic lead halide perovskite nanocrystals. In Nano Tools and Devices for Enhanced Renewable Energy; Devasahayam S., Hussain C. M., Eds.; Elsevier: 2021; pp 61–102.

[ref23] Ashley M. J.; O’Brien M. N.; Hedderick K. R.; Mason J. A.; Ross M. B.; Mirkin C. A. Templated Synthesis of Uniform Perovskite Nanowire Arrays. J. Am. Chem. Soc. 2016, 138 (32), 10096–10099. 10.1021/jacs.6b05901. 27501464

[ref24] Gu L.; Tavakoli M. M.; Zhang D.; Zhang Q.; Waleed A.; Xiao Y.; Tsui K.-H.; Lin Y.; Liao L.; Wang J.; Fan Z. 3D Arrays of 1024-Pixel Image Sensors based on Lead Halide Perovskite Nanowires. Adv. Mater. 2016, 28 (44), 9713–9721. 10.1002/adma.201601603. 27647134

[ref25] Waleed A.; Tavakoli M. M.; Gu L.; Hussain S.; Zhang D.; Poddar S.; Wang Z.; Zhang R.; Fan Z. All Inorganic Cesium Lead Iodide Perovskite Nanowires with Stabilized Cubic Phase at Room Temperature and Nanowire Array-Based Photodetectors. Nano Lett. 2017, 17 (8), 4951–4957. 10.1021/acs.nanolett.7b02101. 28735542

[ref26] Zhang Z.; Suchan K.; Li J.; Hetherington C.; Kiligaridis A.; Unger E.; Scheblykin I. G.; Wallentin J. Vertically Aligned CsPbBr_3_ Nanowire Arrays with Template-Induced Crystal Phase Transition and Stability. J. Phys. Chem. C 2021, 125 (8), 4860–4868. 10.1021/acs.jpcc.0c11217. PMC7976601 33763163

[ref27] Rodová M.; Brožek J.; Knížek K.; Nitsch K. Phase transitions in ternary caesium lead bromide. J. Therm. Anal. Calorim. 2003, 71 (2), 667–673. 10.1023/A:1022836800820.

[ref28] Stoumpos C. C.; Malliakas C. D.; Peters J. A.; Liu Z.; Sebastian M.; Im J.; Chasapis T. C.; Wibowo A. C.; Chung D. Y.; Freeman A. J.; Wessels B. W.; Kanatzidis M. G. Crystal Growth of the Perovskite Semiconductor CsPbBr_3_: A New Material for High-Energy Radiation Detection. Cryst. Growth Des. 2013, 13 (7), 2722–2727. 10.1021/cg400645t.

[ref29] Meng Y.; Lan C.; Li F.; Yip S.; Wei R.; Kang X.; Bu X.; Dong R.; Zhang H.; Ho J. C. Direct Vapor-Liquid-Solid Synthesis of All-Inorganic Perovskite Nanowires for High-Performance Electronics and Optoelectronics. ACS Nano 2019, 13 (5), 6060–6070. 10.1021/acsnano.9b02379. 31067402

[ref30] Gao Y.; Zhao L.; Shang Q.; Zhong Y.; Liu Z.; Chen J.; Zhang Z.; Shi J.; Du W.; Zhang Y.; Chen S.; Gao P.; Liu X.; Wang X.; Zhang Q. Ultrathin CsPbX_3_ Nanowire Arrays with Strong Emission Anisotropy. Adv. Mater. 2018, 30 (31), 1801805 10.1002/adma.201801805. 29923237

[ref31] Chen J.; Fu Y.; Samad L.; Dang L.; Zhao Y.; Shen S.; Guo L.; Jin S. Vapor-Phase Epitaxial Growth of Aligned Nanowire Networks of Cesium Lead Halide Perovskites (CsPbX_3_, X = Cl, Br, I). Nano Lett. 2017, 17 (1), 460–466. 10.1021/acs.nanolett.6b04450. 28002671

[ref32] Oksenberg E.; Sanders E.; Popovitz-Biro R.; Houben L.; Joselevich E. Surface-Guided CsPbBr_3_ Perovskite Nanowires on Flat and Faceted Sapphire with Size-Dependent Photoluminescence and Fast Photoconductive Response. Nano Lett. 2018, 18 (1), 424–433. 10.1021/acs.nanolett.7b04310. 29210586

[ref33] Shoaib M.; Zhang X.; Wang X.; Zhou H.; Xu T.; Wang X.; Hu X.; Liu H.; Fan X.; Zheng W.; Yang T.; Yang S.; Zhang Q.; Zhu X.; Sun L.; Pan A. Directional Growth of Ultralong CsPbBr_3_ Perovskite Nanowires for High-Performance Photodetectors. J. Am. Chem. Soc. 2017, 139 (44), 15592–15595. 10.1021/jacs.7b08818. 29058888

[ref34] Oksenberg E.; Fai C.; Scheblykin I. G.; Joselevich E.; Unger E. L.; Unold T.; Hages C.; Merdasa A. Deconvoluting Energy Transport Mechanisms in Metal Halide Perovskites Using CsPbBr_3_ Nanowires as a Model System. Adv. Funct. Mater. 2021, 31 (22), 2010704 10.1002/adfm.202010704.

[ref35] Shang Q.; Li C.; Zhang S.; Liang Y.; Liu Z.; Liu X.; Zhang Q. Enhanced Optical Absorption and Slowed Light of Reduced-Dimensional CsPbBr_3_ Nanowire Crystal by Exciton-Polariton. Nano Lett. 2020, 20 (2), 1023–1032. 10.1021/acs.nanolett.9b04175. 31917588

[ref36] Ohashi Y.; Yasui N.; Yokota Y.; Yoshikawa A.; Den T. Submicron-diameter phase-separated scintillator fibers for high-resolution X-ray imaging. Appl. Phys. Lett. 2013, 102 (5), 051907 10.1063/1.4790295.

[ref37] Hormozan Y.; Sychugov I.; Linnros J. High-resolution x-ray imaging using a structured scintillator. Med. Phys. 2016, 43 (2), 696–701. 10.1118/1.4939258. 26843233

[ref38] Xu Q.; Guo Y.; Li C.; Wang X.; Li Y.; Zhang B.; Ouyang X. Vertical Nanowires Enhanced Spatial Resolution of X-Ray Imaging. IEEE Photonics Technol. Lett. 2021, 33 (2), 73–76. 10.1109/LPT.2020.3045110.

[ref39] Anttu N.; Xu H. Q. Efficient light management in vertical nanowire arrays for photovoltaics. Opt. Express 2013, 21 (S3), A558–A575. 10.1364/OE.21.00A558. 24104444

[ref40] Mali S. S.; Patil J. V.; Hong C. K. Hot-Air-Assisted Fully Air-Processed Barium Incorporated CsPbI_2_Br Perovskite Thin Films for Highly Efficient and Stable All-Inorganic Perovskite Solar Cells. Nano Lett. 2019, 19 (9), 6213–6220. 10.1021/acs.nanolett.9b02277. 31369285

[ref41] Wu B.; Nguyen H. T.; Ku Z.; Han G.; Giovanni D.; Mathews N.; Fan H. J.; Sum T. C. Discerning the Surface and Bulk Recombination Kinetics of Organic-Inorganic Halide Perovskite Single Crystals. Adv. Energy Mater. 2016, 6 (14), 1600551 10.1002/aenm.201600551.

[ref42] Zhang L.; Zeng Q.; Wang K. Pressure-Induced Structural and Optical Properties of Inorganic Halide Perovskite CsPbBr_3_. J. Phys. Chem. Lett. 2017, 8 (16), 3752–3758. 10.1021/acs.jpclett.7b01577. 28742359

[ref43] Oksenberg E.; Merdasa A.; Houben L.; Kaplan-Ashiri I.; Rothman A.; Scheblykin I. G.; Unger E. L.; Joselevich E. Large lattice distortions and size-dependent bandgap modulation in epitaxial halide perovskite nanowires. Nat. Commun. 2020, 11 (1), 489 10.1038/s41467-020-14365-2. 31980620 PMC6981217

[ref44] Brennan M. C.; Herr J. E.; Nguyen-Beck T. S.; Zinna J.; Draguta S.; Rouvimov S.; Parkhill J.; Kuno M. Origin of the Size-Dependent Stokes Shift in CsPbBr_3_ Perovskite Nanocrystals. J. Am. Chem. Soc. 2017, 139 (35), 12201–12208. 10.1021/jacs.7b05683. 28772067

[ref45] Nikl M.; Yoshikawa A. Recent R&D Trends in Inorganic Single-Crystal Scintillator Materials for Radiation Detection. Adv. Opt. Mater. 2015, 3 (4), 463–481. 10.1002/adom.201400571.

[ref46] Tisseur D.; Estre N.; Tamagno L.; Eleon C.; Eck D.; Payan E.; Cherepy N. Performance evaluation of several well-known and new scintillators for MeV X-ray imaging. In 2018 IEEE Nuclear Science Symposium and Medical Imaging Conference; IEEE: Sydney, Australia, 2018; pp 1–3.

[ref47] Yang B.; Yin L.; Niu G.; Yuan J.-H.; Xue K.-H.; Tan Z.; Miao X.-S.; Niu M.; Du X.; Song H.; Lifshitz E.; Tang J. Lead-Free Halide Rb_2_CuBr_3_ as Sensitive X-Ray Scintillator. Adv. Mater. 2019, 31 (44), 1904711 10.1002/adma.201904711. 31531905

[ref48] Kong X.; Zong K.; Lee S. S. Nanoconfining Optoelectronic Materials for Enhanced Performance and Stability. Chem. Mater. 2019, 31 (14), 4953–4970. 10.1021/acs.chemmater.9b01707.

[ref49] Lin J.-T.; Liao C.-C.; Hsu C.-S.; Chen D.-G.; Chen H.-M.; Tsai M.-K.; Chou P.-T.; Chiu C.-W. Harnessing Dielectric Confinement on Tin Perovskites to Achieve Emission Quantum Yield up to 21%. J. Am. Chem. Soc. 2019, 141 (26), 10324–10330. 10.1021/jacs.9b03148. 31244186

[ref50] Wei J.; Wang Q.; Huo J.; Gao F.; Gan Z.; Zhao Q.; Li H. Mechanisms and Suppression of Photoinduced Degradation in Perovskite Solar Cells. Adv. Energy Mater. 2021, 11 (3), 2002326 10.1002/aenm.202002326.

[ref51] Chen S.; Wen X.; Huang S.; Huang F.; Cheng Y.-B.; Green M.; Ho-Baillie A. Light Illumination Induced Photoluminescence Enhancement and Quenching in Lead Halide Perovskite. Sol. PRL 2017, 1 (1), 1600001 10.1002/solr.201600001.

[ref52] Tian Y.; Peter M.; Unger E.; Abdellah M.; Zheng K.; Pullerits T.; Yartsev A.; Sundström V.; Scheblykin I. G. Mechanistic insights into perovskite photoluminescence enhancement: light curing with oxygen can boost yield thousandfold. Phys. Chem. Chem. Phys. 2015, 17 (38), 24978–24987. 10.1039/C5CP04410C. 26343504

[ref53] Samei E. Image quality in two phosphor-based flat panel digital radiographic detectors. Med. Phys. 2003, 30 (7), 1747–1757. 10.1118/1.1578772. 12906192

[ref54] Farman T. T.; Vandre R. H.; Pajak J. C.; Miller S. R.; Lempicki A.; Farman A. G. Effects of scintillator on the modulation transfer function (MTF) of a digital imaging system. Oral Surgery, Oral Medicine, Oral Pathology, Oral Radiology, and Endodontology 2005, 99 (5), 608–613. 10.1016/j.tripleo.2004.08.013. 15829886

