# Supplementary material for: Single-Crystalline Perovskite Nanowire Arrays for Stable X-ray Scintillators with Micrometer Spatial Resolution
Source: ACS Appl Nano Mater. 2021 Dec 18;5(1):881–9. doi: 10.1021/acsanm.1c03575 (PMC8805114; doi:10.1021/acsanm.1c03575)
Supplement: Supplementary file 1 — an1c03575_si_001.pdf [file an1c03575_si_001.pdf]

Supporting Information

Single-crystalline Perovskite Nanowire Arrays for Stable X-ray Scintillators with Micrometer Spatial Resolution

*Zhaojun Zhang,[1] Hanna Dierks,[1] Nils Lamers,[1] Chen Sun,[2] Klára Nováková,[2] Crispin Hetherington,[3] Ivan G. Scheblykin,[2] Jesper Wallentin[1,]**

[1] Synchrotron Radiation Research and NanoLund, Department of Physics, Lund University, Box 124, Lund, 22100, Sweden

[2] Chemical Physics and NanoLund, Department of Chemistry, Lund University, Box 124, Lund, 22100, Sweden

[3] Centre for Analysis and Synthesis and NanoLund, Department of Chemistry, Lund University, Box 124, Lund, 22100, Sweden

Corresponding author: jesper.wallentin@sljus.lu.se

Experimental section

All the AAO membranes were purchased from Topmembranes Technology Ltd. The AAO membranes provided by the supplier were circular and had two diameters, 13 mm or 25 mm. In Figure 1c, we show a photo of a membrane of 25 mm diameter to demonstrate our growth method can be used for large area uniform growth. For the samples used for characterization, we used an AAO membrane with 13 mm diameter to decrease the cost. The growth procedures were the same for the differently sized membranes.

The 13 mm diameter membranes were treated by O_2 plasma for 2 min to improve the wetting performance of the pores. The glass slide and AAO membrane were cleaned and dried by N_2 gun before use.

The precursor was made by mixing 0.848 g CsBr (Sigma-Aldrich, 99.9%) and 1.468 g $PbBr_2$ (Sigma-Aldrich, 98%) into 10 mL DMSO (Sigma-Aldrich, anhydrous, 99.9%). The precursor concentration was 0.4 mol/L.

First, we put the precursor on the glass slide. Then we put the AAO membrane on the precursor droplet, waited 1-2 min to allow the precursor to infuse into the AAO pores. (For the saturated concentration 0.45M precursor, solvent evaporation at room temperature lead to nucleation within a few minutes. Therefore, we used 0.4M precursor for growth.) The sample was then put on a hotplate and held at 70 ℃. After 1 h the nanowires had formed inside the pores. If we use 5 μL, 10 μL, 20 μL precursor, the grown nanowire length was about 4 μm, 7 μm, 12 μm, respectively. It was important to confine the precursor to the bottom side of the AAO since the volume of the precursor was much larger than the pore volume. If the precursor was allowed onto the top of the AAO, during the evaporation process, it was possible to grow a thin film or polycrystalline grains on top of the AAO surface.

The $CsPbBr_3$ QD film was obtained by drop casting 40 μL $CsPbBr_3$ QD solution (Sigma-Aldrich, 10 mg/mL in toluene) on a glass slide and then heating at 75 ℃ for 20 min.

The $CsPbBr_3$ microcrystal film was grown by a method similar to a previous study[1] but with modifications. First, we put a cleaned glass slide in the beaker, then 1 mL 0.3M $CsPbBr_3$ in DMSO precursor was added to the beaker. 10 ml acetone was then added to the solution drop by drop while keeping the solution stirring at 600 rpm. Finally, the $CsPbBr_3$ was gradually dissolved out. After 10 min precipitation, the glass slide was taken out from the solution. Then the substrate was put on a 70 ℃ hotplate for 10 min followed by a 150 ℃ hotplate for 20 min annealing.

The SEM images were taken with a Hitachi SU-8010 using a voltage of 5 kV and a current of 10 mA. The EDS mapping results were obtained with a GEMI-SEM with an accelerating voltage of 10 kV. The single nanowire for TEM characterization was transferred by using a FIB probe (FEI Nova Nanolab 600) onto the Cu grid. The TEM studies were done on a 200 kV JEOL electron microscope. XRD measurements were acquired using a STOE STADI MP diffractometer with a Cu anode source working at a voltage of 40 kV and a current of 40 mA. The scanning step was 0.2° and the scanning speed was 10 s per step. The detector was a MYTHEN 1K detector which had an angular resolution of 0.03° FWHM constant and the native collected data points had an interval of 0.015°.

The UV laser (378 nm, OXXIUS in CW mode) excited microfocus photoluminescence spectra were collected by a BWTEM Exemplar plus, and the integration time was about 100 ms. The visible light transmission spectra of the sample were collected using an Ocean Optics RED TIDE USB650 spectrometer.

Time-resolved photoluminescence decay measurements were performed using a 485 nm pulse diode laser with a repetition rate in the range 100 Hz-80 MHz. The response time of the detector was less than 200 ps.

The scintillation spectra excited by an X-ray source were collected by an Ocean Optics QE pro spectrometer. The X-ray source was a microfocus X-ray tube Cu target source (Rigaku,

Power: 45 W (45 kV /1 mA)) with a nominal spot size of 20×20 μm. The current was adjustable and had a maximum value of 1 mA. The sample was put 2 cm from the X-ray source. The integration time was 10 s.

The source for the X-ray imaging measurement was the same as the one used for the X-ray luminescence measurement. The JIMA pattern was placed 5.7 cm from the X-ray source, and the scintillator was placed 6.2 cm from the source. The detector was an XSight X-ray CCD detector with a special scintillator free lens unit (manufacturer: Rigaku, Field-of-View (FOV): 1.8mm ×1.35mm, effective pixel size: 0.54 μm). The JIMA pattern used for spatial resolution studies was purchased from Japan Inspection Instruments Manufacturers' Association. The TEM grid was from TED PELLA Inc. To get the MTF function, we used a slant edge method by imaging the edge of a Si Wafer taken from a $CsPbBr_3$ NW/AAO scintillator. The Si wafer surface was aligned with the optical axis via a rotation stage. The X-ray source was operated at 45 kV, 1mA. The total exposure time was 300 s. We also used flat field correction, and tilted the image 1 degree to align the edge with the pixel column.

Table S1: The details of AAO membranes with different pore diameters. The pore distance means the distance between centers of two adjacent pores.

**Pore diameter (nm)**	**30±5**	**60±10**	**90±10**	**170±30**	**250±30**	**360±40**
**pore distance (nm)**	65±5	100±10	125±10	450±20	450±20	450±20
**Membrane thickness (μm)**	50	50	50	50	50	50

Photon yield estimation:

The 500 μm thick YAG:Ce was purchased from (CRYTUR, spol. s r.o.) and has a photon yield of 30000 ph/MeV. Our X-ray source had a Cu target and was operated with a voltage of 45 kV and tube current of about 1 mA. The YAG:Ce scintillator of this thickness absorbs all the X-ray photons. We used an Xpin diode (Rigaku) to measure the absorbed X-ray photons of

all the $CsPbBr_3$ NW/AAO scintillators and the flux of the source. The estimated photon yield is calculated as follows:

$$Photon\ yield_{NW/AAO} = Photon\ yield_{\mathrm{YAG:Ce}} \times \frac{Luminescence_{NW/AAO}}{Luminescence_{\mathrm{YAG:Ce}}} / \frac{Absorbed\ photons}{Source\ photons}$$

Note that the AAO template absorbs a significant share of the X-rays, since the membrane thickness is about 50 µm, which is much thicker than the nanowire length (~7 µm) we used for the scintillation spectra measurement. However, the AAO does not have any scintillation. So, to get the net photon yield of the $CsPbBr_3$ nanowires, we need to compensate for the absorption in the AAO template. We did this by measuring the X-ray photons absorbed by an empty AAO membrane.

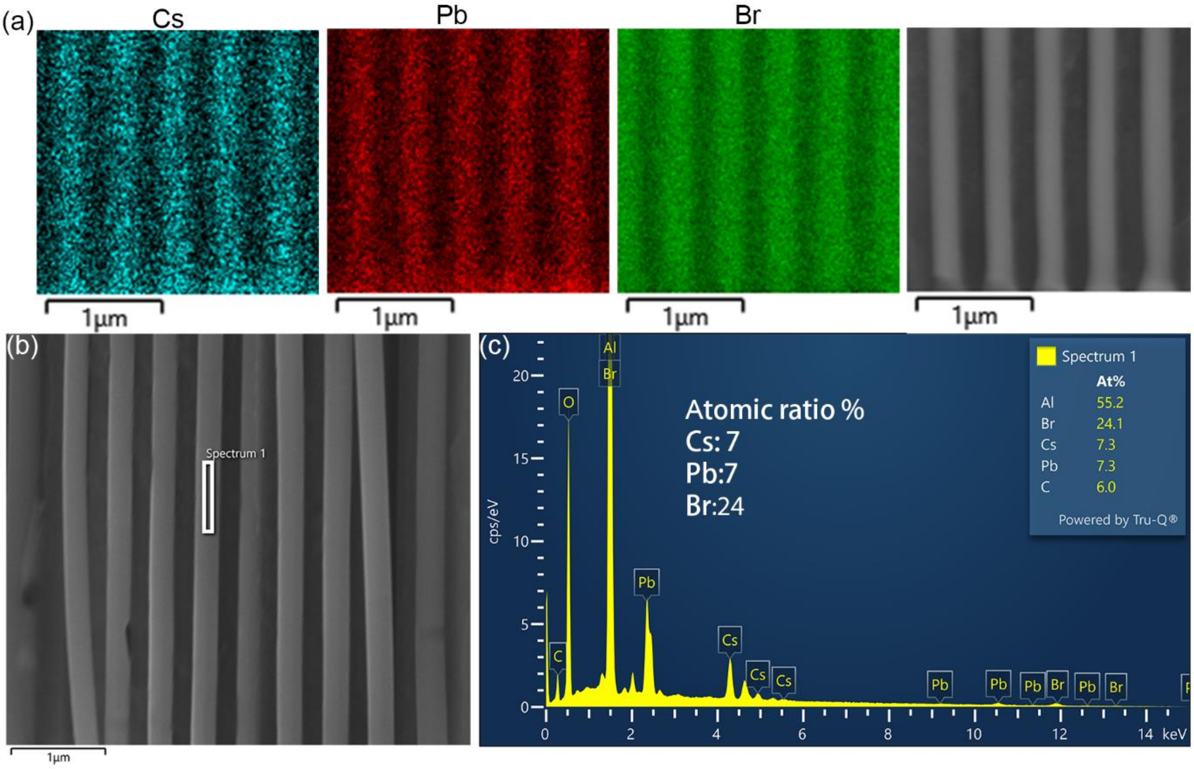

Figure S1: (a) EDS mapping of a cross-section of the $CsPbBr_3$ nanowires inside AAO. (b) SEM image of the EDS element analysis area. (c) The element analysis spectrum of the area in (b) which gives the atomic ratio of Cs:Pb:Br of about 1:1:3.4.

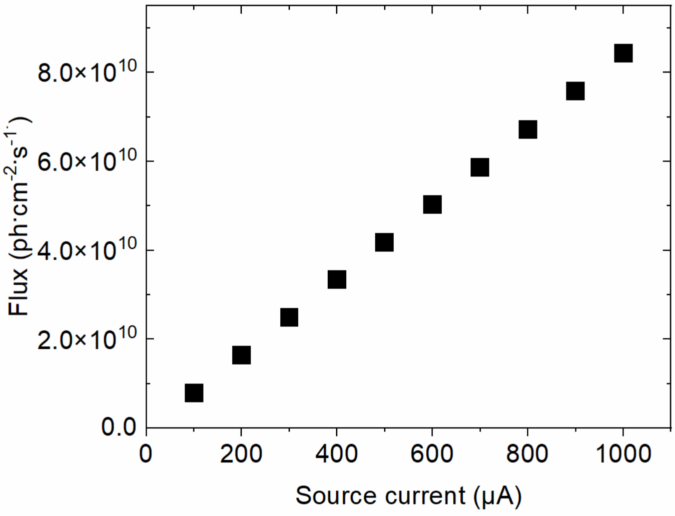

Figure S2: The relation of the X-ray photon flux to the source current.

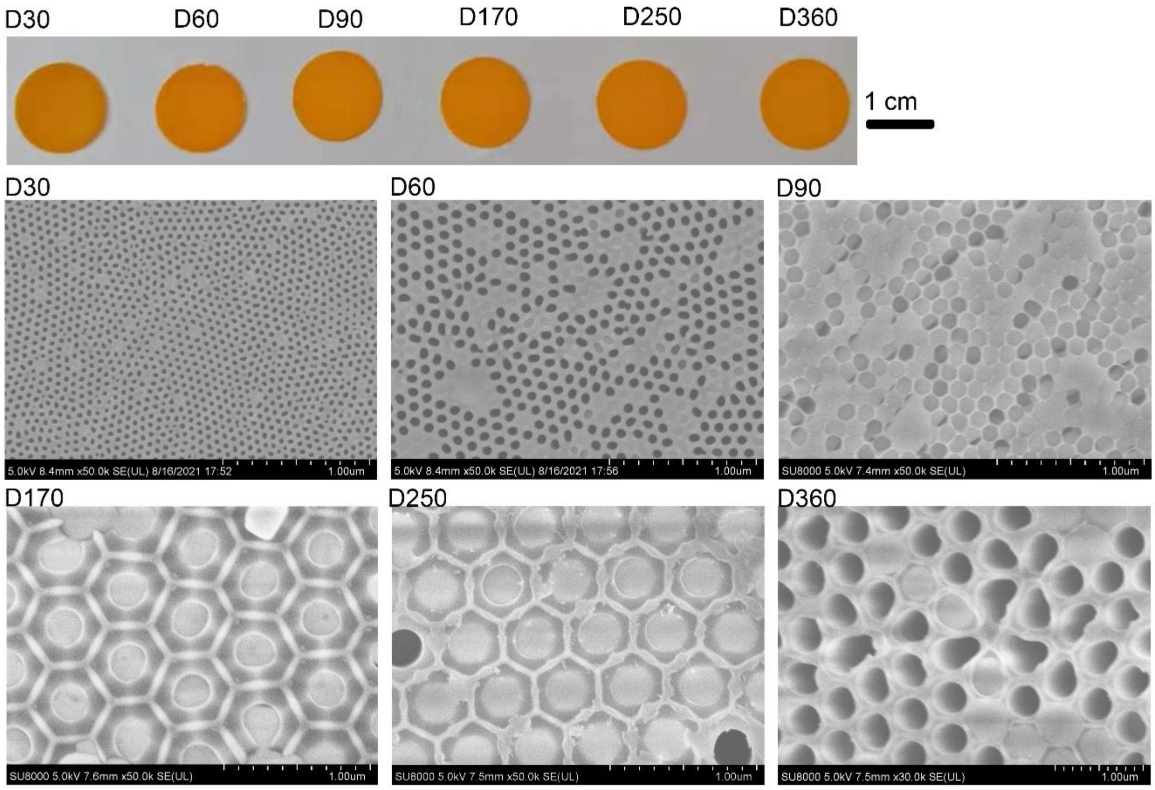

Figure S3: Photo and top view SEM images of $CsPbBr_3$ NW/AAO samples with nanowire diameters of 30 nm, 60 nm, 90 nm, 170 nm, 250 nm, 360 nm.

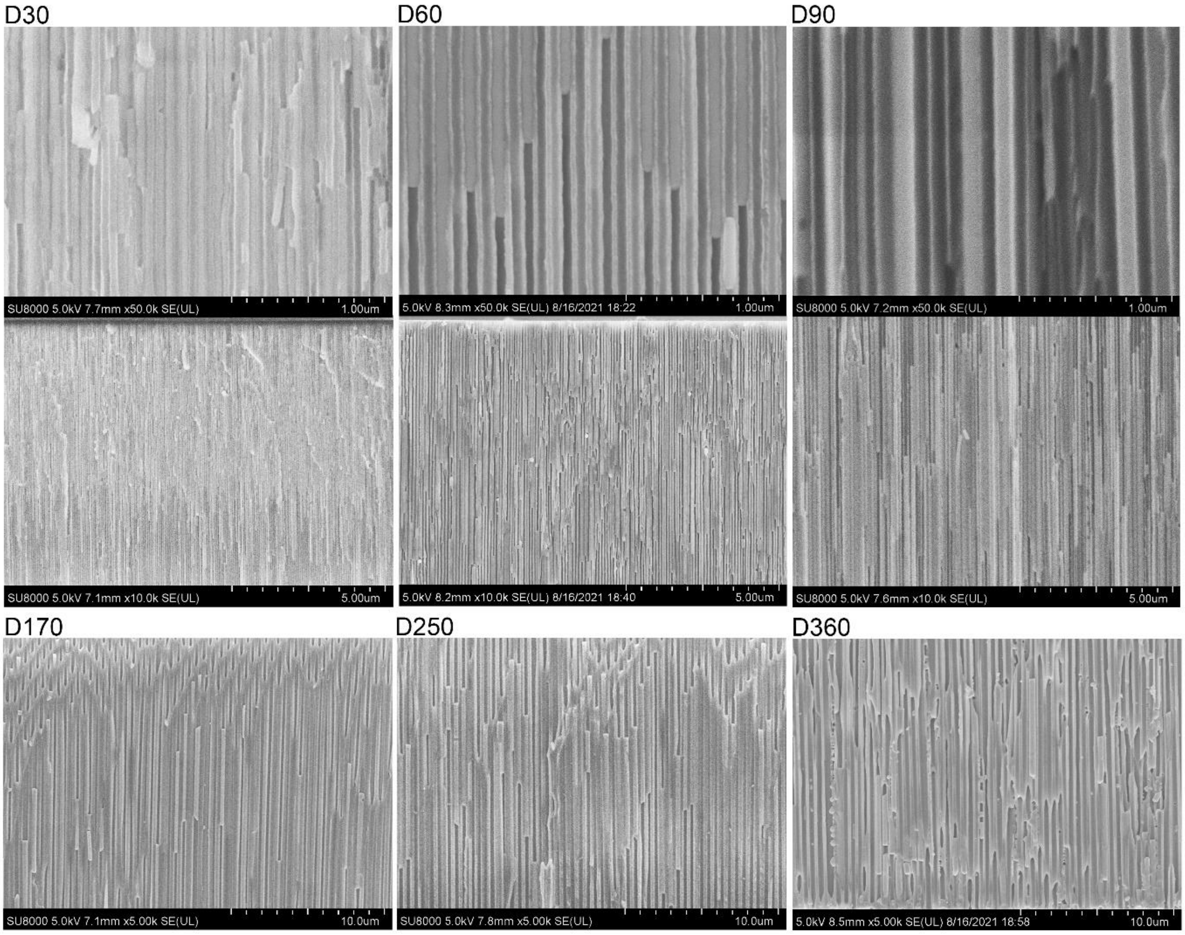

Figure S4: Cross-section SEM images of $CsPbBr_3$ NW/AAO with nanowire diameters of 30 nm, 60 nm, 90 nm, 170 nm, 250 nm, 360 nm.

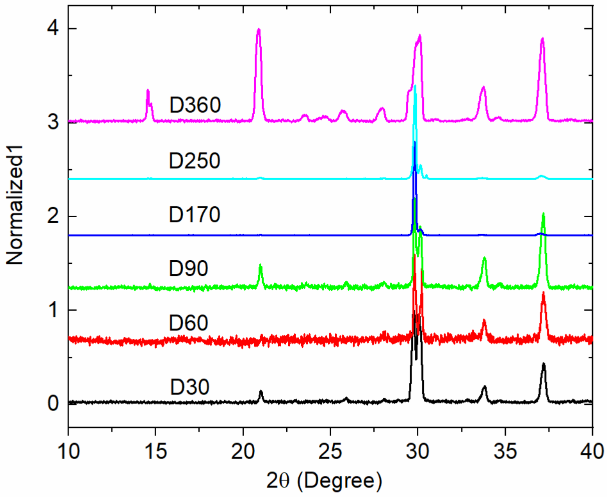

Figure S5: XRD patterns of $CsPbBr_3$ NW/AAO with nanowire diameters of 30 nm, 60 nm, 90 nm, 170 nm, 250 nm, 360 nm.

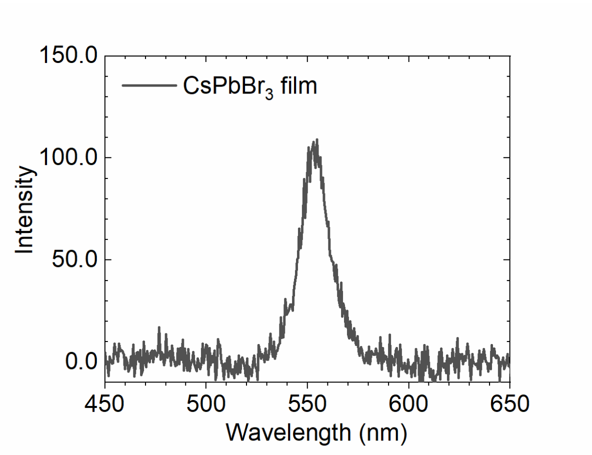

Figure S6: Scintillation spectrum of a 40 μm thick $CsPbBr_3$ microcrystal film under the same measurement conditions as the $CsPbBr_3$ NW/AAO samples.

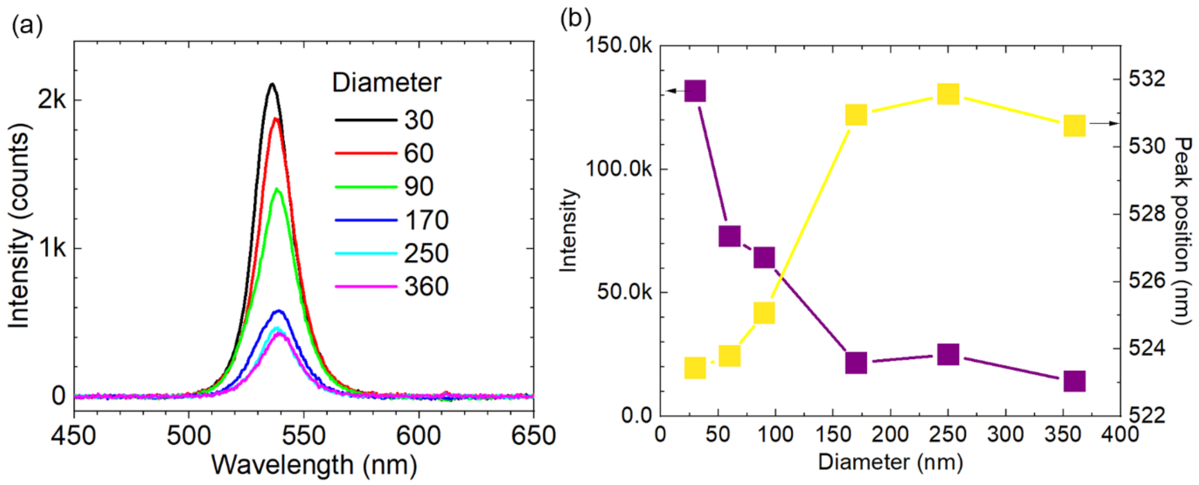

Figure S7: (a) 378 nm laser (CW mode) excited PL spectrum of different diameter $CsPbBr_3$ NWs/AAO (b) Diameter dependence of the fitted integrated luminescence intensity and the peak position.

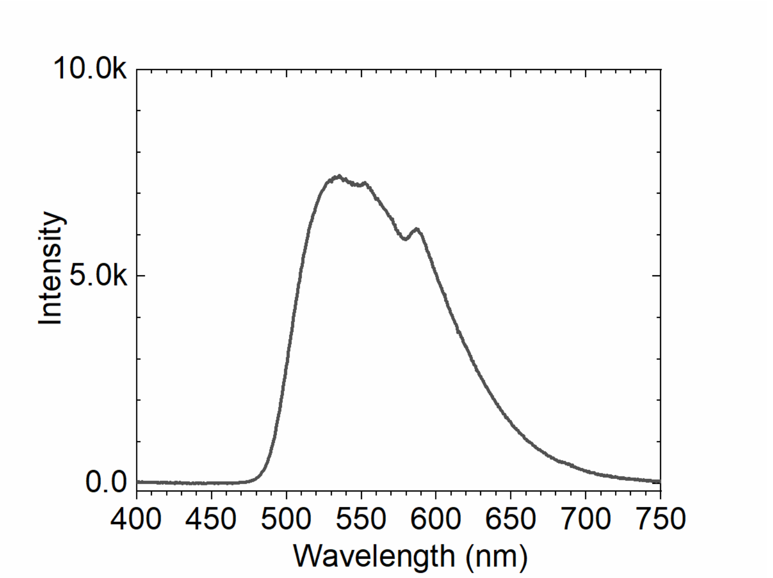

Figure S8: Scintillation spectrum of a commercial 500 μm thick YAG:Ce scintillator under the same measurement conditions as all the other $CsPbBr_3$ NW/AAO scintillators.

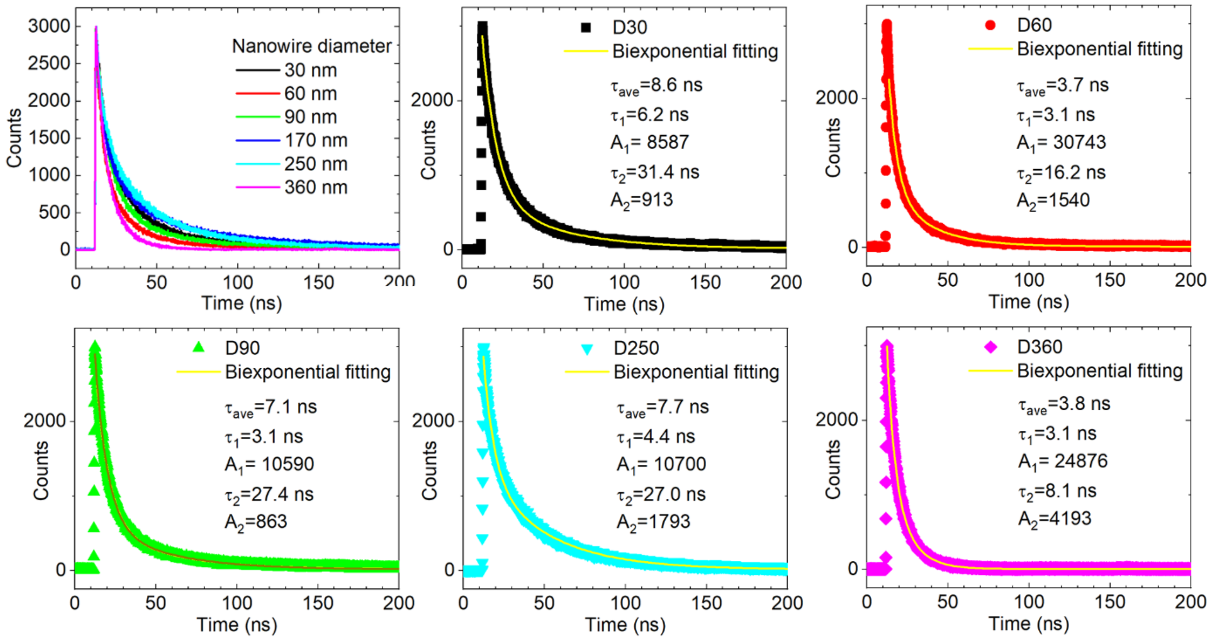

Figure S9: PL lifetime measurements of all the $CsPbBr_3$ NW/AAO samples excited by pulsed 485 nm laser. The biexponential fitting results of D30, D60, D90, D250 and D360 are also shown. The fitting results of D170 sample are shown in Figure 2c.

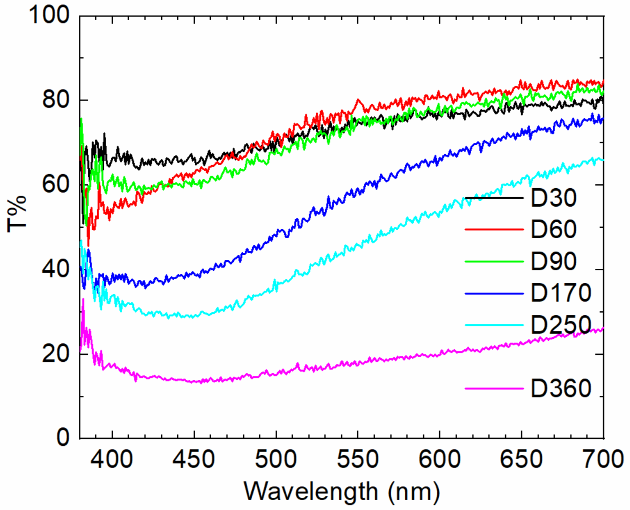

Figure S10: Visible light transmission spectra of empty AAO membranes with different pore diameters.

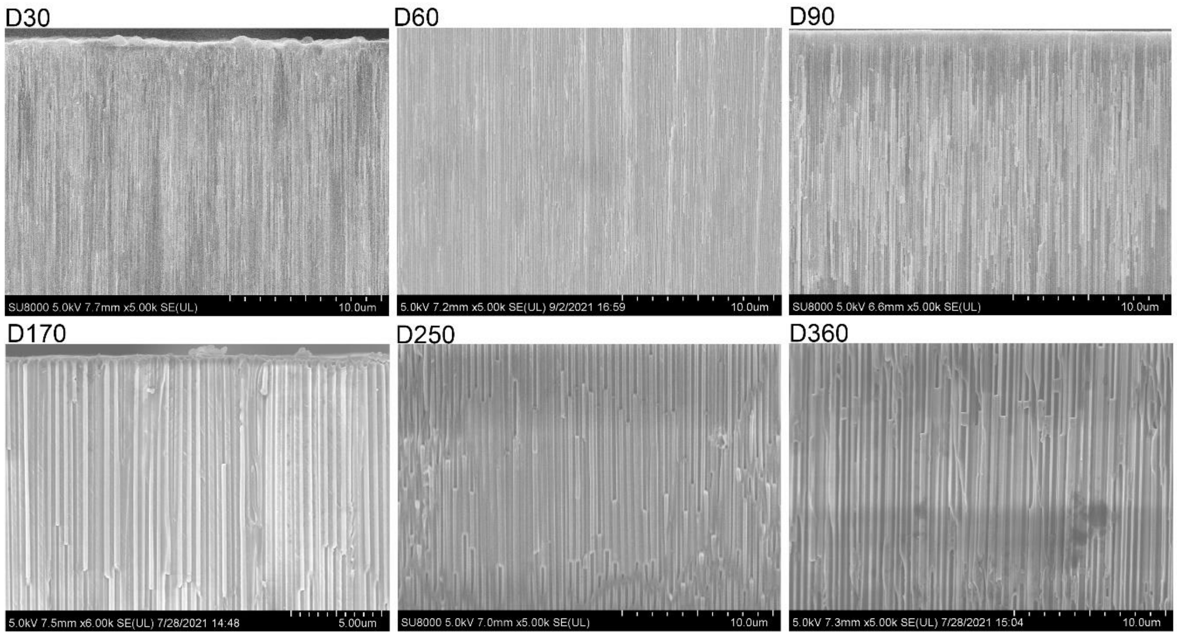

Figure S11: Cross-sectional SEM images of different diameters $CsPbBr_3$ NWs/AAO with nanowire length of about 12-13 μm, which were used for the X-ray imaging spatial resolution measurement.

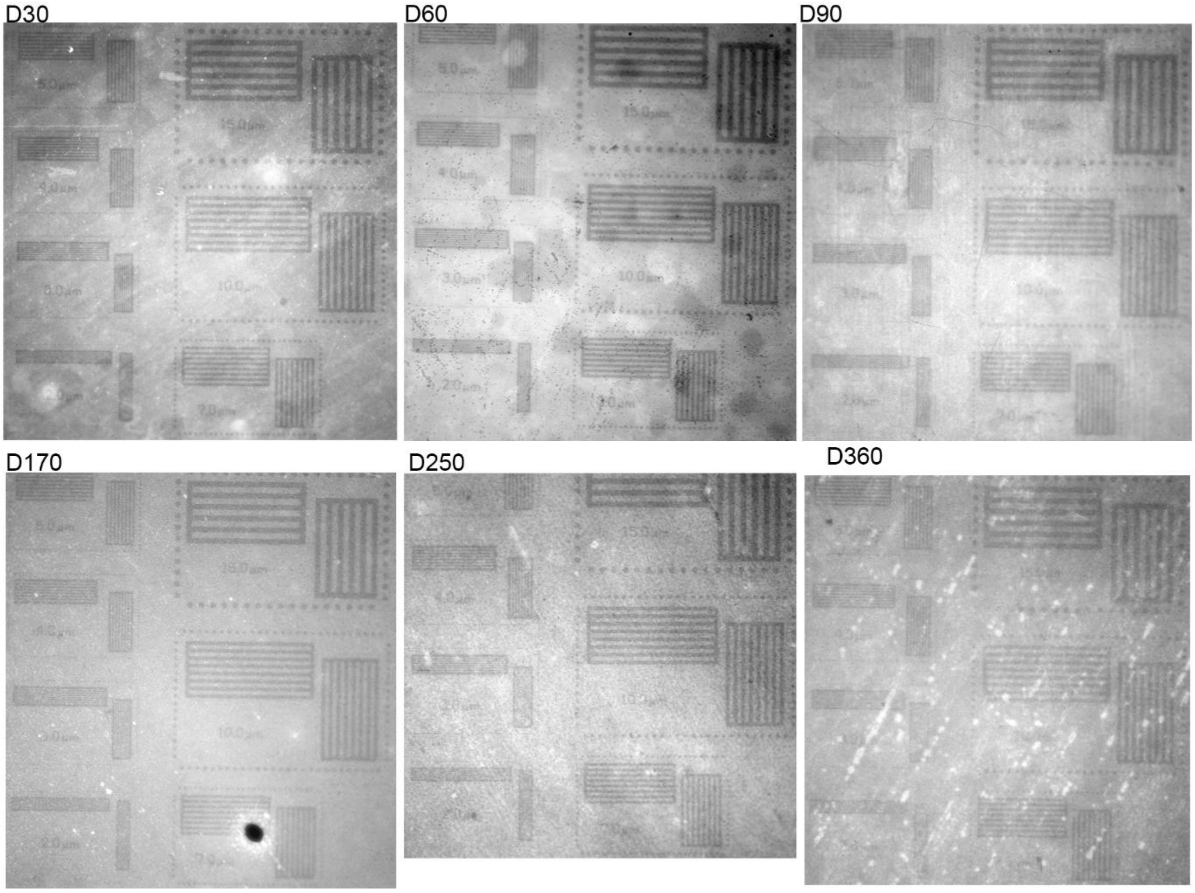

Figure S12: X-ray imaging results for the spatial resolution tests using a JIMA pattern.

References:

1. Gou, Z.; Huanglong, S.; Ke, W., et al., Self-Powered X-Ray Detector Based on All-Inorganic Perovskite Thick Film with High Sensitivity Under Low Dose Rate. *physica status solidi (RRL) – Rapid Research Letters* **2019,** *13* (*8*), 1900094.
